# Successful Management of Pregnancy and Hepatic Toxicity in a CML Female Patient Treated with Nilotinib: a Case Report and a Review

**DOI:** 10.4084/MJHID.2015.020

**Published:** 2015-02-15

**Authors:** Domenico Santorsola, Elisabetta Abruzzese

**Affiliations:** 1 Domenico Santorsola; MD, Servizio Dipartimentale di Ematologia ed Oncologia San Nicola Pellegrino Trani, Italy; 2 Elisabetta Abruzzese, MD, PhD, Hematology, S. Eugenio Hospital, Tor Vergata University, Rome, Italy

## Abstract

We report a case of a young patient with chronic viral hepatitis HBV infection, diagnosed with CML in March 2006 and treated with imatinib 400mg/die as first line therapy with concomitant Lamivudine. Patient obtained a complete hematologic response (CHR) in 2 months, complete cytogenetic response (CCyR) in six months and major molecular response (MMR) at 24 months. After three years of treatment, she became imatinib intolerant and resistant. In November 2009 patient started nilotinib 400mg/BID. Patient tolerated well the new molecule never experiencing hepatic impairment. After switching to nilotinib, she reached in 12 months transcript reduction more than 3 log (MMR). Even if patient had been informed of the need of continuous therapy and to use effective methods of contraception during tyrosine kinase inhibitor (TKI) treatment, in 2012 she decided to plan a pregnancy. In August 2012 a MR^4^ was documented, and treatment discontinued before starting pregnancy. She was placed on interferon and observed throughout her pregnancy. The disease remained stable achieving an undetectable transcript level; she delivered a healthy boy in September 2013. Treatment with nilotinib was re-started three months after delivery, and she is still in molecular remission (MR^5^). A complete discussion of the case and the available literature is presented.

## Introduction

Imatinib, the first BCR–ABL1 tyrosine kinase inhibitor (TKI) approved for the treatment of patients with chronic myeloid leukemia (CML), has profoundly changed the management of CML improving the prognosis and the quality of life for such patients.^1^,^2^ The second and third generation TKIs include Nilotinib (Tasigna, Novartis), Dasatinib (Sprycel, Bristol Myers Squibb), Bosutinib (Bosulif, Pfizer), and the recently approved Ponatinib (Iclusig, Ariad Pharma).

Results with imatinib at 8 years are excellent with rates of complete cytogenetic remission (CCyR) of 82%, and an estimated overall survival of 89%, but 1/3 of patients need to change treatment mainly due to adverse events (AE)/intolerance or unsatisfactory therapeutic outcome.^3^ Nilotinib, as other second generation TKIs, is a safe and efficient treatment for long-term use in patients with chronic phase (CP) CML, who are intolerant of, or resistant to imatinib.^4^

We report a 34-year-old woman with CML and HBV-infection, who became imatinib resistant/intolerant successfully reaching deep molecular response with nilotinib as a second line therapy. Her chronic viral hepatitis B infection was managed without reactivation, and she got successfully pregnant after nilotinib discontinuation.

## Case Report and Literature Review

In April 2006, patient was diagnosed with CP-CML with classic (9;22) (q34, q11) translocation found in all 20 metaphases and a BCR-ABL p210 b2a2 transcript, intermediate Sokal risk. Past medical history was unremarkabkle, except for a HBV-positive infection diagnosed in 1990.

In May 2006 she started treatment with imatinib 400mg/die. Patient also started the antiviral therapy with Lamivudine that was well tolerated and stopped on July 2006 for sustained negative viral load. The patient achieved complete hematologic response (CHR) within two months and CCyR in six months. From November 2006 to July 2007 patient underwent several imatinib dose reduction and suspension due to persistent leucopenia grade 3 while maintaining a CCyR. After 24 months (June 2008) patient achieved major molecular response (MMR). In December 2008 patient achieved a MR^4^.

Unfortunately, after achieving MMR, patient became again imatinib intolerant this time by experiencing severe dermatological toxicity that was critically affecting her quality of life (QoL) and did not resolve after dose reduction and local therapy. For this, in November 2009 patient stopped imatinib and started nilotinib 400mg/BID as second line therapy.

Biochemical laboratory abnormalities were described using Nilotinb, including elevations of alanine aminotransferase and aspartate aminotransferase, but no cross-intolerance to skin reactions were reported.^5^ Since starting nilotinib, the patient has been strictly monitored every two weeks for all hepatic functionality markers with no abnormalities recorded and no reactivation of HBV. The patient regained MMR in 12 months and completely recovered from the dermatological toxicity dramatically improving her QoL.

**Figure 1:**
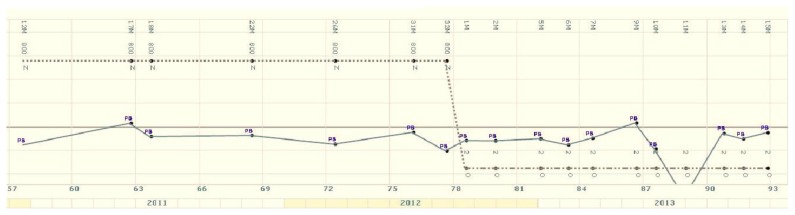
Molecular response monitoring. Molecular results diagram International Scale from december 2010 to december 2013 (Labnet italian network laboratory). The gray line is the MMR line. The dashed “....” line indicates Nilotinib therapy, line drops at interruption (July 2012).

Even if patient had been informed of the need of continuous therapy and to use effective methods of contraception since data on pregnancy during TKIs treatment were limited in 2012, she decided to plan a pregnancy. At this time, molecular response was stable (Figure 1).

It is mandatory to stop TKI when a pregnancy in a female patient on therapy is planned or just started, due to the teratogenic potential of all TKIs.^6^

So in July 2012, after having confirmed three stable molecular results patient discontinued nilotinib and started interferon with 3 MU sc. three times a week. In August 2012 a BCR–ABL1 transcript reduction more than 4 log (< MR^4^) was documented. Patient and her husband started their pregnancy plan with all necessary gynecological, and andrologic assessments and patient got pregnant on December 31^st^ 2012. After all necessary evaluation, patient has been consent for the collection and preservation of umbilical cord blood, which show no presence of CML transcript. Patient was observed through the whole pregnancy period; the disease remained stable achieving also in one sample an undetectable transcript level. On September 1^st^, 2013 patient delivered an healthy boy after 42 weeks of gestation with a natural childbirth.

At birth baby weight was 3.65 Kg., length 52 cm; the baby is healthy, normally growing and just turned a little more than 1 year old.

Treatment with nilotinib was re-initiated three months after delivery since patient expressed the desire to breastfeed that was judged not contraindicated during interferon therapy. After nilotinib restart, patient confirmed her molecular remission with MR^5^ undetectable transcript. Patient restarted therapy in agreement with his hematologist since there were no trials available at this time on TKIs interruption, and did not want to continue with Interpheron, neither was feeling safe in stopping therapy.

## Discussion

Imatinib and the subsequent second and third generation TKIs are recognized to represent the major advancement in clinical research leading to a successful targeted therapy with substantial improvement of survival and quality of life in CML patients.

Considering the significant proportion of female/male patients diagnosed with CML in reproductive age, and the lifespan of responding patients, issues relating to fertility and pregnancy are often requested by patients. For this reason, nowadays the management of fertility should begin at diagnosis. A patient in reproductive age should be informed about the risk of unplanned pregnancies in terms of foetal problems and/or the risk of a loss of response/progression after stopping therapy to carry the pregnancy. However, it is important to inform that a pregnancy can be conducted when the treatment has being started, and the response is optimal.^7^

Limited data are reported concerning either female/male pregnancies conception while on nilotinib therapy. A recently published complete and updated review on TKIs and pregnancy of all literature refers to 46 male and 3 female on nilotinb getting pregnant.^8^ This should be the 4^th^ case.

In the same review suggestions for the management of a conception/pregnancy, while on TKIs, are reported. No particular risks of fertility for male patients taking imatinib or nilotinib are present, and conception and pregnancy outcomes have been evidenced. Caution should be used during dasatinib due to the little data available and the wide range substrate inhibition (other than TKIs) of this drug.^9^ No reports are available for patients taking bosutinib or ponatinib. For those patients, the possibility to cryopreserve sperm before starting therapy should be discussed.

In a female patient in reproductive age, effective contraception should be suggested at diagnosis. A pregnancy should be only planned after the milestone of a stable MMR, or better (>MR4.5) has been reached from at list 18–24 months. Obgyn visit for preconception tests (including in some cases male sperm evaluation), ultrasound and planned conception is highly recommended. Therapy should be stopped immediately before or right after conception. All TKIs must be avoided during the organogenesis (post menstrual days 31–71, weeks 5–13). Q-PCR must be monitored each month/2 months to follow the transcript, depending on the molecular results.

Therapy during pregnancy should be only considered if a cytogenetic or hematologic relapse occur. Each case should be individually evaluated taking into account the rapidity of the relapse, the clinical history of the patient, and most of all the pregnancy status (weeks of gestation).

Interferon can be considered safe before and during pregnancy;^10^ hydroxyurea can be used to control leucocytosis after organogenesis.^11^ If necessary, considering the little passage of imatinib and nilotinib in the fetal compartment, TKIs therapy can be considered after placenta has being formed, and organogenesis completed.^12^ Since dasatinib pass the placenta, it should be avoided throughout all the pregnancy.^13^

After delivery therapy can be postponed to consent breast feeding, provided a low level of molecular transcript, or according to the haematologist judgement. If it is necessary to resume treatment, patient can breast-feed the baby the first 2–5 days post-partum to give him the colostrums.^8^

Although this experience is limited to a single patient, the success of the outcome demonstrates that the management of chronic myeloid leukemia in women with childbearing potential should be individualized. Based on the incoming results of the treatment-free remission (TFR) studies, drugs can be successfully stopped during pregnancy and the same therapy resumed afterwards. A sustained profound molecular response is an essential entry criterion for maintaining MMR. All patients were sensitive to TKI retreatment.^14^ Several other TKIs discontinuation studies are on-going and may serve to provide a new treatment paradigm for younger patients who will benefit of pregnancy without exposure to TKIs.
